# YB-1 dependent oncolytic adenovirus efficiently inhibits tumor growth of glioma cancer stem like cells

**DOI:** 10.1186/1479-5876-11-216

**Published:** 2013-09-18

**Authors:** Klaus Mantwill, Ulrike Naumann, Janina Seznec, Vroni Girbinger, Hermann Lage, Pawel Surowiak, Dagmar Beier, Michel Mittelbronn, Jürgen Schlegel, Per Sonne Holm

**Affiliations:** 1 Institut für Experimentelle Onkologie & Therapieforschung, Klinikum rechts der Isar, Technische Universität München, Ismaninger Str. 22, 81675 München, Germany; 2 Laboratory of Molecular Neuro-Oncology, Department of Vascular Neurology, Hertie Institute for Clinical Brain Research, University of Tübingen, Tübingen, Germany; 3 Institute of Pathology, Charité, Berlin, Germany; 4 Department of Neurology, Aachen, Medical School, Aachen, Germany; 5 Institute of Neurology, Edinger Institute, Goethe-University Frankfurt, Frankfurt, Germany; 6 Division of Neuropathology, Institute of Pathology, Technische Universität München, Munich, Germany; 7 XVir Therapeutics GmbH, Munich, Germany

**Keywords:** Cancer stem cells, YB-1, Virotherapy, Oncolytic virus, Temozolomide

## Abstract

**Background:**

The brain cancer stem cell (CSC) model describes a small subset of glioma cells as being responsible for tumor initiation, conferring therapy resistance and tumor recurrence. In brain CSC, the *PI3-K*/AKT and the RAS/*mitogen activated protein kinase* (MAPK) pathways are found to be activated. In consequence, the human transcription factor YB-1, knowing to be responsible for the emergence of drug resistance and driving adenoviral replication, is phosphorylated and activated. With this knowledge, YB-1 was established in the past as a biomarker for disease progression and prognosis. This study determines the expression of YB-1 in glioblastoma (GBM) specimen *in vivo* and in brain CSC lines. In addition, the capacity of Ad-Delo3-RGD, an YB-1 dependent oncolytic adenovirus, to eradicate CSC was evaluated both *in vitro* and *in vivo*.

**Methods:**

YB-1 expression was investigated by immunoblot and immuno-histochemistry. *In vitro*, viral replication as well as the capacity of Ad-Delo3-RGD to replicate in and, in consequence, to kill CSC was determined by real-time PCR and clonogenic dilution assays. *In vivo*, Ad-Delo3-RGD-mediated tumor growth inhibition was evaluated in an orthotopic mouse GBM model. Safety and specificity of Ad-Delo3-RGD were investigated in immortalized human astrocytes and by siRNA-mediated downregulation of YB-1.

**Results:**

YB-1 is highly expressed in brain CSC lines and in GBM specimen. Efficient viral replication in and virus-mediated lysis of CSC was observed *in vitro*. Experiments addressing safety aspects of Ad-Delo3-RGD showed that (i) virus production in human astrocytes was significantly reduced compared to wild type adenovirus (Ad-WT) and (ii) knockdown of YB-1 significantly reduced virus replication. Mice harboring othotopic GBM developed from a temozolomide (TMZ)-resistant GBM derived CSC line which was intratumorally injected with Ad-Delo3-RGD survived significantly longer than mice receiving PBS-injections or TMZ treatment.

**Conclusion:**

The results of this study supported YB-1 based virotherapy as an attractive therapeutic strategy for GBM treatment which will be exploited further in multimodal treatment concepts.

## Background

GBM is among the most deadly human cancers. Despite modern diagnosis and improved treatment regimens, including surgical resection followed by radiation and chemotherapy with TMZ, the prognosis for patients with GBM remains poor with a median survival after diagnosis of less than 15 months [1,2]. Thus, new GBM therapeutic strategies are desperately needed. Considerable research efforts have been focused on dissecting the role of cancer stem cells (CSC) also referred to as tumor initiating cells (TIC), in cancer progression and recurrence [3,4]. CSC have been described in several tumor types, including GBM [5,6]. A number of studies have explored their role in overall tumor treatment resistance producing contradictory results [7-9]. Still, the detailed mechanisms of treatment resistance have to be characterized. Nevertheless, it is currently believed that CSCs are responsible for tumor initiation, progression and relapse, and that depletion of these cells is obligatory to cure patients.

On the transcriptional and protein level several signaling pathways, including PI3-K/AKT and the RAS/MAPK pathway, have been identified in brain CSC [10-12]. One of the downstream phosphorylation substrates of both pathways is YB-1, a multifunctional protein regulating transcription and translation [13]. However, apart from these important results very little is known about the expression of YB-1 in CSC. The mouse homologue YB-1 is widely expressed throughout early mouse development, including neural tube closure and cell proliferation, but is barely detectable in normal differentiated cells [14]. In contrast, YB-1 is highly expressed in cancer cells, and an increasing number of scientific articles have left little doubt that YB-1 promotes tumor growth and drug resistance [15,16]. Hence, YB-1 has been shown to be a relevant biomarker for clinical outcome of cancer patients [17-19]. Recently, Dunn and colleagues found a link between YB-1 and breast tumor initiating cells. They reported that YB-1 induces breast cancer tumor initiating cells to express CD44 and CD49f leading to enhanced cell growth and drug resistance [20]. Thus, we hypothesized that YB-1 is highly expressed in CSC derived from GBM, too. This assumption is also supported by the finding that the transcription factor Twist, directly involved in generating a breast cancer stem cell phenotype, is highly expressed in GBM [21], and promotes tumor cell growth through YB-1 expression [22]. Moreover, several essential signaling pathways which are activated in CSC, including *signal transducer and activator of transcription* (STAT)3, *nuclear factor kappa B* (NFκB), PKB/AKT and MAPK/ERK are known to target YB-1 [13,23].

Viruses that replicate selectively in tumor cells but not in normal cells are used as agents to fight cancer. This therapeutic approach is known as virotherapy [24]. Various oncolytic viruses have displayed potential to efficiently kill not only cancer cells, but also CSC [25,26]. We have previously described the oncolytic adenovirus (OAV) Ad-Delo3-RGD which was rendered cancer-specific by deletion of the transactivation domain CR3 of the E1A13S protein. This deletion restricts viral amplification and anti-tumor activity to drug-resistant cells displaying nuclear YB-1 expression [27]. In addition, Ad-Delo3-RGD contains an E1B19-deletion and a RGD-modified fiber. In a recent study, we have demonstrated the anti-GBM efficacy of Ad-Delo3-RGD in combination with TMZ both *in vitro* and *in vivo*[28].

Based on this knowledge and combined with the observation that high YB-1 expression and/or its nuclear localization are closely associated with poor prognosis in GBM and other malignancies [29,30], we hypothesize that nuclear YB-1 protein expression due to activated PI3-K/AKT and the RAS/MAPK pathways is significantly elevated in brain CSC, and thus may be useful in ablating CSC by Ad-Delo3-RGD. In the present study we have now analyzed YB-1 protein expression in brain CSC and non-neoplastic tissue. In addition, we examined the capacity of an YB-1 based virotherapy approach in eradicating brain CSC *in vitro* and *in vivo* in a TMZ-resistant GBM-CSC model.

## Methods

### Cell culture

U87-MG (ATCC), U373-MG and LN-18 cells (kindly provided by Dr. N. de Tribolet, Zurich, Switzerland) were maintained in DMEM with glutamine (Biochrom, Berlin, Germany) containing 10% FCS (PAN-Biotech, Aidenbach, Germany). Brain CSC lines R11, R28, R40, and R49 were obtained from patients with primary GBM as previously described [31] and were maintained as tumorspheres in stem cell-permissive DMEM-F12 medium supplemented with 20 ng/ml of each human recombinant epidermal growth factor (EGF; BD Biosciences, Heidelberg, Germany), human recombinant basic fibroblast growth factor (bFGF; R&D Systems, Wiesbaden, Germany), human leukemia inhibitory factor (LIF; Millipore, Billerica, MA, USA), and 2% B27 (Life Technologies, Carlsbad, CA, USA) for preservation of the tumors’ original molecular characteristics and for minor differentiation. SV-GA cells (a human astrocytic subclone of human fetal glial cells transduced with an origin-defective mutant of simian virus 40) have been previously described [32] and were maintained in MEM medium with 2 mM L-glutamine, 10% fetal bovine serum, and antibiotic solution.

### Adenoviral vectors

The following viruses were used: (i) wild type adenovirus of serotype 5 (Ad-WT), (ii) Ad-Delo3-RGD, an oncolytic adenovirus (OAV) that combines the dl520 genotype (CR3 deletion of E1A restricting viral amplification and anti-tumor activity to drug-resistant cells displaying nuclear YB-1 expression) with an E1B19K deletion and a RGD motif in the fiber knob [27], (iii) dl703 [33] that contains expanded deletions in early region 1 (3180 bp) and (iv) the E1A-deleted adenovirus dl312 described in detail in [34]. All viruses were produced in HEK293 cells and purified by two consecutive standard cesium chloride gradient centrifugations and size-exclusion chromatography (PD-10 Desalting Columns, GE Healthcare, Freiburg, Germany). Viral titers were determined by plaque assay using HEK293 cells. Multiplicity of infection (MOI) is therefore indicated as plaque forming units (pfu) per cell. Virus dose was optimized for each *in vitro* experiment. The absence of replication competent adenovirus (RCA) in Ad-Delo3-RGD preparations was excluded by PCR using specific primers for the E1A-CR3 region (for primer sequences proceed to “DNA isolation and PCR”). In general, particle (determined by OD-measurement) to PFU ratio in virus preparation were between 30 and 50.

### Treatment with temozolomide

To calculate the EC_50_ of TMZ (Schering-Plough, Kenilworth, NJ, USA) in brain CSC lines, the cells were separated by trituation and viability was assessed by trypan blue staining. 10.000 viable brain CSC were treated with increasing concentrations of TMZ (1–2000 μM) for 24 h followed by a medium change. After further 72 h, cell viability was assessed using the MTT assay.

### Immunoblot analysis

Cells were lysed using ProteoJET Mammalian Cell Lysis Reagent (Fermentas, St. Lon-Rot, Germany) supplemented with complete protease inhibitor cocktail (Roche Diagnostics, Filderstadt, Germany) and incubated at room temperature for 10 min. The lysates were clarified by centrifugation and protein concentration was measured using the Bradford assay. 40 μg protein was separated on SDS-polyacrylamide gels and transferred onto poly-vinylidine-diluloride (PVDF) membranes (Millipore, Schwalbach, Germany). For detection, the following antibodies were used: rabbit anti-phospho-YB1 (Ser102), rabbit PathScan^®^ Multiplex Western Cocktail I (anti-phospho-p90RSK, anti-phospho-AKT, anti-phospho-p44/42 MAPK (Erk1/2), anti-phospho-S6 Ribosomal Protein Detection Kit), rabbit anti-phospho-AKT (all antibodies were purchase from Cell Signaling/Millipore), goat anti-MGMT (R&D Systems) or rabbit anti-YB-1 [27]. Immunoreactive proteins were detected using the Amersham enhanced chemiluminescence (ECL) or ECL plus western blot detection system (GE Healthcare).

### DNA isolation and polymerase chain reaction

For the assessment of viral replication, total DNA from infected cells (50 MOI) was isolated using digestion buffer (100 mM NaCl, 10 mM TrisHCl pH 8.0, 25 mM EDTA pH 8.0, 0.5% SDS), Proteinase K and phenol-chloroform. After precipitation with ethanol, DNA was solubilized in 10 mM TrisHCl pH 8.0. Quantitative real-time PCR was performed using the ABI Prism 7900HT sequence detection system (Applied Biosystems) using 100 ng of total DNA per reaction and SYBR green fluorescent dye (Agilent Technologies, Waldbronn, Germany). The specific primers (Eurofins, Hamburg, Germany) used for real-time PCR analyses were: fiber-fw: 5′-AAGCTAGCCCTGCAAACATCA, fiber-rev: 5′-CCCAAGCTACCAGTGGCAGTA, ß-actin-fw: 5′-TAAGTAGGTGCACAGTAGGTCTGA, and ß-actin-rev: 5′-AAAGTGCAAAGAACACGGCTAAG. For identity testing following primer were used: 12Sfw: 5’-AATGGCCGCCAGTCTTTT, 12Srev: 5’-GCCATGCAAGTTAAACATTATC, 13Sfw: 5’-GGCATGTTTGTCTACAGTAAG, 13Srev: 5’-GCCATGCAAGTTAAACATTATC, E1b19kfw: 5’-CGTGAGAGTTGGTGGGCGT, E1b19krev: 5’-CTTCGCTCCATTTATCCT, E3ADPfw: 5’-ATGTCAGCATCTGACTTTGGCC, E3ADPrev: 5’-CTCGAGGAATCATGTCTC, E3fw: 5’-GTTAATGTCAGGTCGCCTAAGTCG, E3rev: 5’-GTGTGTTGCCCGCGACCATT RGDfw: 5’-CTGCCGCGGAGACTGTTTC, RGDrev: 5’-CTGCAATTGAAAAATAAACACG. Cycling conditions started with initial enzyme activation at 95°C for 15 min, followed by 40 cycles of 15 sec denaturation at 95°C, 15 sec, annealing at 60°C, and 15 sec elongation at 72°C. Homogeneity of the amplification product was confirmed by melting curve analysis (T_m_-fiber: 85°C, T_m_-ß-actin: 83°C). Detection of amplification of the adenoviral sequences E1A12S, E1A13S, E1B19, E3, ADP and RGD (35 cycles, annealing at 55°C) was done by agarose gel electrophoresis.

### Immunocytochemistry and immunohistochemistry

Brain CSC were grown on slides and fixed for 20 min in a methanol/acetone (1:1) mixture at −20°C. Immunocytochemical reactions were performed using rabbit anti-YB-1 antibody followed by a FITC-conjugated swine anti-rabbit secondary antibody (1:20; Dako, Hamburg, Germany). Slides were mounted with Vectashield (Vector Laboratories/Axxora, Lörrach, Germany) and images were taken with the AxioImagerZ1 with ApoTome (Zeiss Opticals, Jena, Germany). Immunohistochemical reactions were conducted using polyclonal rabbit antibodies directed against YB-1 as previously described [35].

### Hypoxia and clonogenic dilution assay

R11, R28 or R40 cells were seeded in 12-well plates (1 × 10^5^ cells in 0.5 ml stem cell medium per well) and infected with the indicated viruses the next day. Cells were cultivated for further 24–36 h under normoxic or hypoxic (<0.66% O_2_) conditions [36]. After this treatment, cells from 12-well plates were diluted for the clonogenic dilution assay into 24-well plates containing 1ml of stem cell medium in 1:10 dilution steps and incubated for approximately 10 doubling times (4–6 weeks).

### Inhibition of YB-1 by siRNA

1.5 x 10^6^ R28 cells were transfected with 1000 pmol annealed double-strand control-siRNA (Qiagen, Hilden, Germany; sense 5’-UUCUCCGAACGUGUCACG UdTdT-3’, antisense 5’-ACGUGACACGUUCGGAGAAdTdT-3’)’ or a siRNA specific for YB-1 (Qiagen; sense 5’-GGCGAAGGUUCCCACCUUATT-3’, antisense 5’-UAAGGUGGGAACCUUCGCCTG-3), using Lipofectamine™ 2000 (Life Technologies). After 24 h, cells were divided into three aliquots and infected with 50 MOI dl703, Ad-Delo3-RGD or Ad-WT, respectively. Cells were harvested after 4 h and after 48 h. DNA was isolated as described above and solubilized in 10 mM TrisHCl pH 8.0. Expression of YB-1 was confirmed in cells harvested 48 h after siRNA transfection using immunoblot.

### Intracranial tumor model

NMRI nude mice (Janvier, Le Genest Saint Isle, France) were anesthetized and placed into a stereotactic fixation device (Stoelting, Wood Dale, USA). 10^5^ viable R28 cells were injected into the right striatum. At day 7 post implantation, the mice were randomly separated in 4 groups (n=7 to 8 mice per group), followed by an intratumoral injection of either PBS (mock) or 3 x 10^8^ plaques forming units( pfu) Ad-Delo3-RGD. Half of the mice were treated intraperitoneally with TMZ (5 mg/kg) at day 10 and 17. The mice were sacrificed when developing neurological symptoms. Brains were isolated and fixed with 4% paraformaldehyde for further analysis. All animal research was carried out in accordance with the German Animal Welfare Act and was approved by local authorities.

### Statistical analysis

If not other mentioned, figures show representative data from at least three independent experiments. Quantitative data were assessed using *t*-test. To estimate the potency of Ad-Delo3-RGD in the animal GBM-CSC model, Kaplan-Meier curves were prepared and log-rank analysis was performed using SPSS16.0 (IBM, Stuttgart, Germany). All p-values given are unadjusted, two-sided and subjected to a significance level of 5% (* p=0.05; ** p=0.01; *** p=0.0001).

### Histopathological analysis

Tumors of mice were dissected, fixed in 4% formaldehyde, and embedded into paraffin. Serial 5 μm sections were cut and stained with hematoxylin and eosin (H&E). Histopathological evaluations were done on a light microscope (Eclipse E200, Nikon Instruments, Düsseldorf, Germany).

## Results

### Brain CSC lines express activated AKT and MAPK/ERK

**Figure 1:**
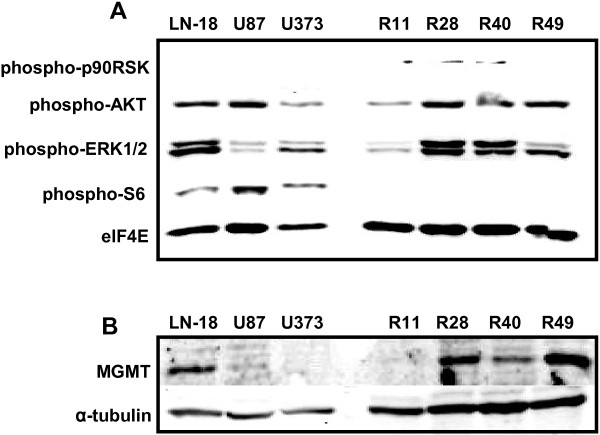
**Brain CSC lines (R11, R28, R40, R49) display activated AKT/MAPK pathway and differential MGMT expression. (A)** Immunoblot of GBM cell lines (LN-18, U87-MG, U373-MG) and brain CSC lines (R11, R28, R40, R49) using the Multiplex PathScan antibody cocktail. ElF4E served as loading control. **(B)** Immunoblot showing MGMT expression. Tubulin served as a loading control. Brain CSC lines showed differential expression of MGMT. Of the established GBM cell lines, only LN-18 showed MGMT expression. R28 cells showed high MGMT expression and were used for the orthotopic mouse GBM model.

It is established that AKT, MAPK/ERK and ribosomal S6 kinase (RSK) can interact with and phosphorylate YB-1 [37-39]. To explore the presence of activated AKT, MAPK/ERK and RSK in brain CSC, we examined protein phosphorylation by immunoblot analysis (Figure 1A). All four brain CSC lines showed phospho-AKT and phospho-ERK expression to a different degree. Especially striking was the phosphorylation of RSK in R11, R28 and R40 CSCs in comparison to established GBM cell lines (U87-MG, U373-MG, LN-18). In LN-18 cells which are characterized by high O^6^-Methylguanin-DNA-Methyltransferase (MGMT) expression and TMZ resistance [40], phospho-AKT and phospho-ERK_1/2_ are equally expressed as it is the case for the brain CSC lines R28 and R40. In contrast to established GBM cell lines, brain CSC lines showed no expression of phospho-S6.

### MGMT is differentially expressed in brain CSC lines

TMZ resistance was reported in brain CSC [8], and different responsiveness to treatment with TMZ according to the MGMT status of these cells was described [9]. Since our set of brain CSCs showed different grades of TMZ resistance (high in R28, low in R11, intermediate in R40 and R49; being EC_50_ in all CSC higher than the clinical therapeutic TMZ concentration of ~50 μM; data not shown), we tested MGMT expression in these cells using immunoblot analysis. We also included the GBM cell line LN-18 known to express MGMT and being highly resistant to TMZ [40] as well as U87-MG and U373-MG cells in this analysis. As shown in Figure 1B, MGMT was detectable in LN-18, R28, R40 and R49 cell lines, but not in U87-MG, U373-MG or R11 cells.

### Brain CSC lines show high expression of YB-1

**Figure 2:**
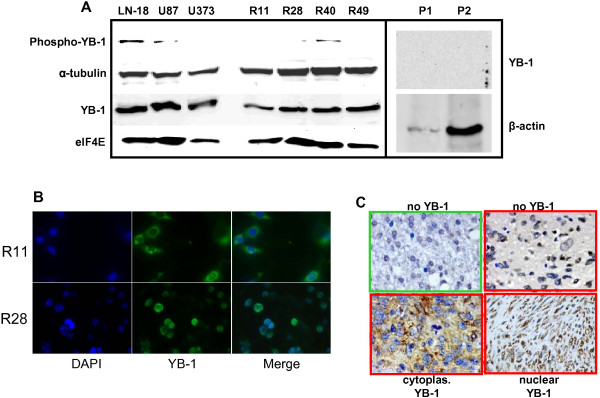
**YB-1 is expressed and phosphorylated in human brain CSC lines, but not in normal CNS tissue. (A)** Immunoblot of YB-1 and phospho-YB-1. ElF4E, α-tubulin and β-actin served as loading controls. Normal brain tissue specimens were obtained from two patients (P1 + P2) without any pathological central nervous system alterations. The amount of protein in P1 was rarely sufficient to detect phospho-YB-1 or considerable amounts of β-actin. **(B)** Immunocytochemistry of R11 and R28 cells demonstrated predominantly cytoplasmic (R11) or nuclear (R28) expression of YB-1, respectively. Blue color: DAPI staining of nuclei. Green color: YB-1 staining. **(C)** Immunohistochemical staining using polyclonal antibodies directed against YB-1 (brown). All tissues were counterstained with hematoxyline (blue). Upper left (green box): non-neoplastic brain tissue did not exhibit any YB-1 expression. Tissues prepared from GBM patients (red boxes) showed varying expression patterns. Upper right: no YB-1 expression, lower left: cytoplasmic YB-1 expression, lower right: nuclear YB-1 expression.

Considering YB-1 as a downstream target of PI3-K/AKT and MAPK/RSK signaling, we next evaluated YB-1 expression in normal brain tissue, GBM cell as well as brain CSC lines. Western blot analysis demonstrates elevated levels of both total and phosphorylated YB-1 in brain CSC lines and established GBM cell lines. In contrast, virtually no YB-1 expression was detectable in normal CNS tissue (Figure 2A right panel and Figure 2C, upper left micrograph). Localization of YB-1 varied in the examined brain CSC lines: In the TMZ-resistant brain CSC line R28, YB-1 is highly phosphorylated and located in the nucleus, whereas in R11 cells, showing the lowest IC_50_ value for TMZ, YB-1 is predominately located in the cytoplasm (Figure 2B), corresponding to the weak phosphorylation status of YB-1 these cells. As shown in Figure 2C, immunohistochemical analysis of non-neoplastic brain tissue demonstrated no expression of YB-1. In glioma specimen, diversifying YB-1 expression patterns could be observed. A few glioma showed no YB-1 expression whereas in the majority of glioma biopsies, YB-1 could be easily detected. Some glioma exhibited cytoplasmic YB-1 expression whereas in other GBM specimen nuclear YB-1-specific staining could be demonstrated.

### YB-1 promotes high level replication of Ad-Delo3-RGD in brain CSC lines

**Figure 3:**
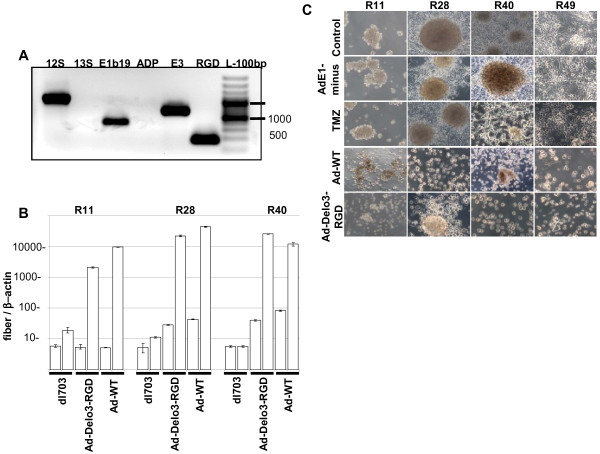
**Ad-Delo3-RGD replicates in and kills brain CSC lines similarly to wild type adenovirus. (A)** PCR analysis of Ad-Delo3-RGD preparation demonstrates the absence of E1A13S sequences and for this the absence of RCA in purified Ad-Delo3-RGD. **(B)** PCR-based assessment of virus replication of an E1A-deleted adenovirus (dl703), Ad-Delo3-RGD or Ad-WT in R11, R28 and R40 CSC. Ad-Delo3-RGD replicates around 130 fold (R11) or 345 fold (R28) better in CSC than dl703. In R40 brain CSC line, Ad-Delo3-RGD replicates around 620 fold better than dl703, and even 4.3 fold better than Ad-WT. Viral DNA for PCR amplification was isolated from infected cells 4 h (white bars) or 72 h (grey bars) post infection. Values are given as relation of adenoviral fiber DNA copies to β-actin copies. Columns represent the mean of 3 measurements **(C)** Tumorspheres of brain CSC lines are destroyed by the YB-1 dependent adenovirus Ad-Delo3-RGD. Brain CSCs were seeded in 6-well plates and 24 h later infected with 50 MOI of the indicated virus, or were treated with 100 μM TMZ. 5–7 days later morphology was studied microscopically (magnification = 100 x). Control: untreated CSC spheres.

We have shown that YB-1 plays an important role in the adenoviral life cycle [41]. In addition, we have proven that drug-resistant cells displaying nuclear YB-1 expression facilitate adenovirus replication independent of the adenoviral E1A protein [42]. Thus, it seems reasonable to assess the replication capacity of an YB-1 dependent adenoviral vector in brain CSC. To exclude contamination of Ad-Delo3-RGD preparation with an YB-1-independent replication competent adenovirus (RCA), we analyzed all Ad-Delo3-RGD preparations for absence of E1A13S sequences which are essential for YB-1 independent virus replication [27]. As demonstrated in Figure 3A, no amplification of adenoviral DNA was measured using primers specific for E1A13S. To analyze whether Ad-Delo3-RGD was able to replicate in brain CSC, we infected these cells with a E1-deleted, replication deficient adenovirus (dl703), Ad-WT or Ad-Delo3-RGD and measured viral DNA replication by real-time PCR 4 h and 72 h post infection. As shown in Figure 3B, Ad-Delo3-RGD replicates around 100–600 fold better than dl703, and in R40 cells even 4.3 fold better than Ad-WT, which shows slightly better replication in the two other CSC lines (R11, R28). Under hypoxic conditions Ad-Delo3-RGD retains its ability to replicate (data not shown). Taken together, these data demonstrate that Ad-Delo3-RGD replicates efficiently in brain CSC.

### Assessment of adenoviral oncolysis in brain CSC *in vitro*

**Figure 4:**
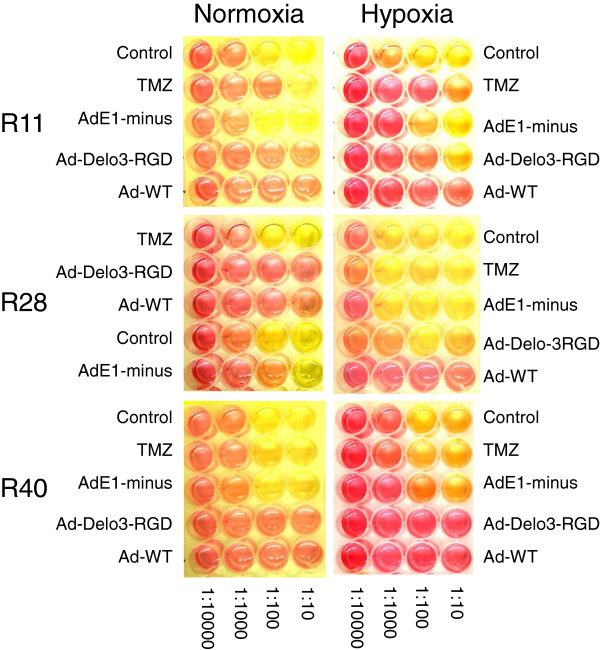
**Ad-Delo3-RGD efficiently kills brain CSC lines under normoxic and hypoxic conditions.** In a clonogenic dilution assay, adenovirally infected CSC were diluted in 1:10 steps in stem cell-permissive DMEM-F12 medium and incubated for 4–6 weeks until the medium turned yellow due to metabolic acidification. Control: untreated; TMZ: temozolomide [100 μM]; AdE1-minus: replication-deficient adenovirus dl703; Ad-WT: wild type adenovirus; Ad-Delo3-RGD: YB-1 dependent oncolytic adenovirus. Red color indicates no metabolic activity due to low cell survival whereas yellow color indicates high metabolic activity due to treatment failure, indicating cell survival. A representative experiment is shown.

To investigate whether Ad-Delo3-RGD induces a virus based cytopathic effect (CPE) in infected brain CSC lines, CPE assays were performed. As shown in Figure 3C, Ad-Delo3-RGD infection resulted in nearly complete cytolysis of infected brain CSC lines within 7 days, which was confirmed by MTT assay (data not shown). In contrast to Ad-Delo3-RGD infected brain CSC, in R28 and R49 cells expressing high levels of MGMT morphology was not altered by treatment with TMZ. In addition, infection of all CSC lines with the replication deficient adenovirus dl703 did not show any lytic effect. To confirm our results we additionally performed cell clonogenic dilution assays with infected brain CSC lines. As shown in Figure 4 (left panel), treatment of brain CSC lines with Ad-Delo3-RGD considerably inhibited growth in a sustained manner (indicated by the red color of the medium six weeks post infection). In contrast, TMZ treated or dl703 infected brain CSC, even under hypoxic conditions (Figure 4, right panel), retain their capacity to grow and of being metabolically active (leading to a yellow color of the medium six weeks post infection).

### Inhibition of YB-1 by siRNA reduces viral replication

**Figure 5:**
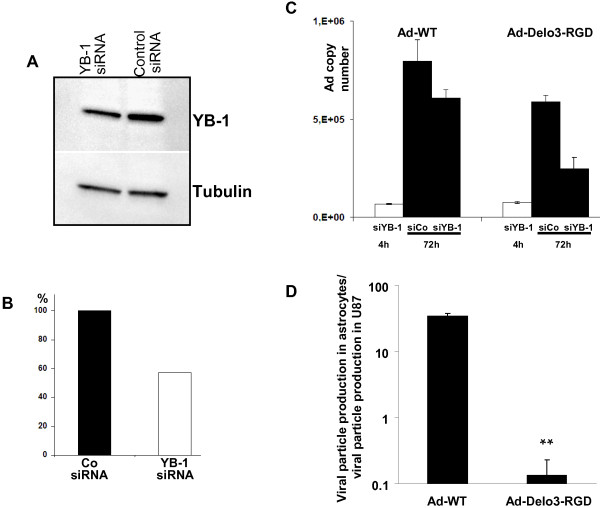
**siRNA mediated downregulation of YB-1 reduced viral replication in infected CSC. (A)** Immunblot of YB-1 in R28 cells 48 h after transfection with either control- or YB-1 specific siRNA. Tubulin serves as a loading control. **(B)** Quantification of YB-1 expression in R28 knockdown cells (normalized to tubulin). **(C)** R28 cells were transfected with control siRNA (siCo) or siRNA against YB-1 (siYB-1), respectively. 48 h later, cells were infected with 50 MOI of either Ad-WT or Ad-Delo3-RGD. Cells were harvested and DNA was isolated 4 h or 72 h after infection. Adenoviral DNA copy numbers as a degree of viral replication were assessed using real-time PCR. siRNA mediated knockdown of YB-1 by reduced Ad-WT replication in infected R28 cells to 76% and Ad-Delo3-RGD-replication to 42%. **(D)** Ad-Delo3-RGD does not replicate efficiently in human immortalized astrocytic SV-GA cells. Data represent the final viral titer as pfu/ml. Viral titers were determined by hexon titer assay from lysates of infected U87MG and SV-GA cells. 175.000 cells were infected with 10 MOI of Ad-WT or Ad-Delo3-RGD under serum-starved conditions (0.5% fetal bovine serum, no growth supplements) for 20 min. In the case of equal particle formation in both cell lines the ratio is = 1. Thus, a ratio >1 indicates higher viral production in astrocytes compared to U87MG cells. A ratio < 1 indicates lower viral production in astrocytes compared to U87MG cells (n=3, ** p=0.0028,)

The impact of YB-1 in adenoviral replication was verified by siRNA-mediated knockdown of YB-1 in R28 cells. Down regulation of YB-1 was demonstrated by immunoblot 48 h post transfection. YB-1 was down regulated to 58% in YB-1 siRNA transfected R28 cells (Figure 5A,B). Knockdown of YB-1 in R28 cells resulted in reduced adenoviral replication compared to control siRNA transfected R28 cells (Figure 5C). 48 h after infection, copy numbers of adenoviral vectors in YB-1 knockdown R28 cells decreased to 76% (Ad-WT) and 42% (Ad-Delo3-RGD) in comparison to copy numbers in control siRNA transfected cells.

### Replication profile of Ad-Delo3-RGD in human astrocytes

Based on the observation that nuclear YB-1 expression is a prerequisite of Ad-Delo3-RGD replication we suggested that Ad-Delo3-RGD will be unable to replicate in human non-dividing non-neoplastic brain cells. To confirm this hypothesis, we infected human immortalized SV-GA astrocytic cells with 10 MOI of Ad-Delo3-RGD or Ad-WT and compared particle formation in these cells in comparison to U87MG glioma cells. Ad-WT replication was even better in SV-GA cells compared to U87MG glioma cells (33-fold increase), but replication of the oncolytic adenovirus Ad-Delo3-RGD was markedly and significantly reduced in SV-GA cells compared to U87MG cells (Figure 5D).

### High therapeutic impact and good safety profile of Ad-Delo3-RGD in a mouse orthotopic TMZ resistant GBM/CSC brain tumor model

**Figure 6:**
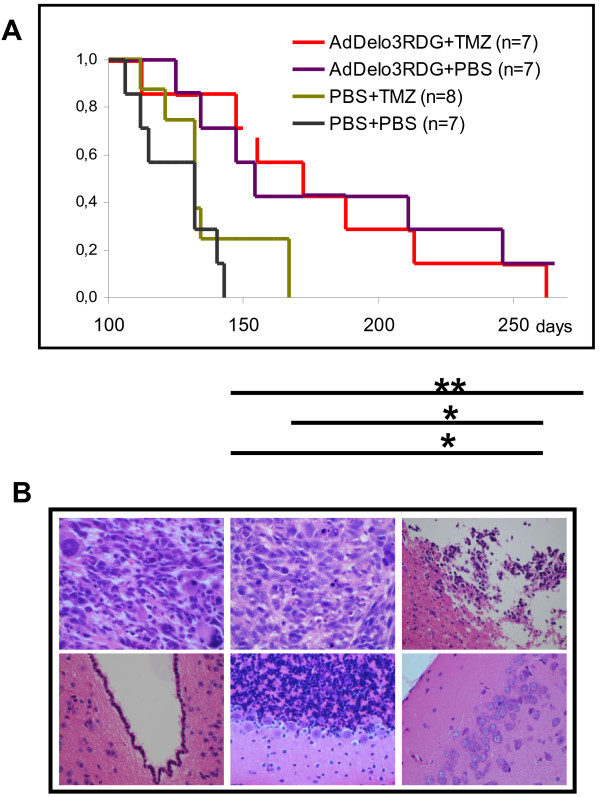
**R28-GBM bearing mice treated with Ad-Delo3-RGD showed prolonged survival without any sign of toxicity. (A)** Survival data of R28 CSC-derived GBM bearing mice treated as indicated are plotted using the Kaplan-Meier method. p-values were determined using the log-rank test. R28-GBM bearing mice treated with Ad-Delo3-RGD or Ad-Delo3-RGD plus TMZ survived significantly longer than mice treated with TMZ or PBS alone. (PBS vs. Ad-Delo3-RGD, p = 0.007; PBS vs. TMZ + Ad-Delo3-RGD, p = 0.013; TMZ vs. Ad-Delo3-RGD, p = 0.023; TMZ vs. TMZ + Ad-Delo3-RGD, p = 0.035. **(B)** Histopathological analyses of tumors (upper panel) and of non-neoplastic tumor adjacent brain tissue of Ad-Delo3-RGD-treated mice (lower panel). Upper left: PBS-treatment; upper middle: TMZ-treatment; upper right Ad-Delo3-RGD-injection. Lower panel: Ad-Delo3-RGD shows no toxicity in any non-neoplastic part of the brain including subventricula zone (left), cerebellum (middle) and cortex (right). 5 μm sections were stained with H&E.

To evaluate the potential of Ad-Delo3-RGD in affecting GBM growth, we established a mouse orthotopic brain tumor model using TMZ-resistant R28 brain CSC cells. These cells express high levels of MGMT (Figure 1B) as well as of *aldehyde dehydrogenase* (ALDH) 1A1 [43], both proteins procuring resistance to TMZ. R28-derived GBM-bearing mice were treated with either TMZ, were intratumorally injected with Ad-Delo3-RGD or achieved a combination therapy. The two groups treated with Ad-Delo3-RGD (either with or without additional TMZ treatment) survived significantly longer than mice which achieved solely TMZ or were mock-(PBS)-treated (p < 0.001). Median survival time increased from 125 days in mock treated mice up to 176 days in Ad-Delo3-RGD treated mice. 167 days post treatment none of the mice receiving an intratumoral PBS (mock) injection or were treated solely with TMZ (0/15) survived, whereas 50% (7/14) of the mice receiving an intratumoral Ad-Delo3-RGD injection were still alive. TMZ treatment of mice did not show any significant effect in none of the treatment groups (Figure 6A). Histopathological evaluation of mock-treated tumors (Figure 6B, upper left) as well as of tumors of TMZ treated mice (Figure 6B, upper middle) showed a similar histopathological appearance. In these mice, polymorphic, highly mitotic tumor tissue was detectable. In contrast, tumors of mice achieving intratumoral Ad-Delo3-RGD injections were smaller, presenting high numbers of apoptotic cells and less vital tumor cells (Figure 6B, upper right). However, in virus treated animals no toxicity or inflammation could be observed in any non-neoplastic part of the brain adjacent to the tumor including the subventricular zone (Figure 6B, lower left), cerebellum (Figure 6B, lower middle) and cortex (Figure 6B, lower right).

## Discussion

It is well established that GBM show increased activation of different signaling pathways including PI3-K/AKT and MAPK/ERK. Signaling through both ERK and AKT is implicated in drug resistance and cell invasion [44,45]. The same has been described for CSC of the brain. Drug resistance and invasive growth are features that make this tumor so difficult to treat. Beside this, identification of CSC in GBM has been found to be of prognostic value [46,47].

Several lines of evidence have indicated a close relationship of YB-1 function and PI3-K/AKT and MAPK/ERK mediated signaling in tumor cells, including direct phosphorylation of YB-1 (serine^102^) by AKT and by RSK, a downstream player of the MAPK signaling cascade, thereby affecting cellular localization and biological function of YB-1 [13,48]. YB-1 in its function as a transcription factor regulates gene expression by binding to promoter regions containing a Y-box motif. Amongst others, YB-1 activates gene expression of the *epidermal growth factor receptor* (EGFR), *matrix metalloproteinase 2* (MMP-2) and of the receptor tyrosine kinase c-MET, all this associated with tumor cell adhesion, invasion and metastasis. Thus, YB-1 could be positioned as a key player in the PI3-K/AKT and MAPK pathways [49]. Mentionable is also the observation, that YB-1 expression is regulated by Twist, which in turn is transcriptionally induced by STAT3. Both are known to play an important role in epithelial to mesenchymal transition (EMT), maintenance of cancer initiating cells and multidrug resistance [50,51].

The importance of YB-1 in conferring multidrug resistance is well documented [13,16]. The role of YB-1 in cancer initiation has, until recently, not been investigated. Dunn and colleagues have shown that blocking YB-1 protein expression delayed tumor onset in mice. In addition, they demonstrated that YB-1 is involved in tumor initiating surface marker expression, including CD44 in breast cancer initiating cells. Based on these findings, they postulate that MAPK/RSK phosphorylation and activation of its downstream targets, including YB-1, promote a cancer initiating phenotype [20].

In a first step, we examined the above mentioned pathways, which turned out to be generally activated in brain CSC (Figure 1A). Next, we studied the downstream target YB-1. We found considerable expression as well as phosphorylation and therefore activation of YB-1 in all brain CSC and GBM cell lines analyzed so far, but not in non-neoplastic brain tissue (Figure 2). This is in line with previous studies that evaluated YB-1 expression in pediatric primary GBM and non-neoplastic brain tissue [29]. However, even if the major fraction of GBM expresses YB-1, its expression level and subcellular localization varies among tumors of different patients (Figure 2C). Since it is known that YB-1 will be up-regulated as well as activated by phosphorylation and nuclear localization in patients who initially have been treated with radio-chemotherapy, the detected variability of YB-1 expression in different GBM specimen may be a result of different chemotherapy approaches and different cycles of chemotherapy the patients received. This fact makes Ad-Delo3-RGD treatment of patients with recurrent GBM, who achieved chemo-radiotherapy, an interesting virotherapeutic strategy, since notably these patients will present high amounts of activated YB-1 in their tumor cells.

On the one hand, YB-1 like Twist is capable to induce EMT in some tumor entities [52] but on the other hand, EMT has been reported to be linked to the gain of epithelial stem cell properties [53]. In addition, migrating GBM cells showing a stemness-like phenotype are characterized by expressing high levels of CD44 and low levels of *programmed cell death protein* (PDCD)4, a factor known to inhibit YB-1 expression [54]. Taken together, our data support the idea of Dunn and colleagues that YB-1 might promote a cancer initiating phenotype. However, the role of YB-1 in brain CSC tumorigenicity remains to be studied in detail and was beyond the scope of this work.

It has been reported that embryonal carcinoma stem cells support adenoviral replication more efficiently than differentiated derivatives, hypothesizing that a cellular factor with E1A-like activity is regulated during differentiation in stem cells [55]. We have previously reported that the recombinant adenovirus Ad-Delo3-RGD, containing a certain deletion in the E1A gene, replicates in nuclear YB-1 positive cancer cells [27]. We used Ad-Delo3-RGD which contained a RGD motif to increase infectivity in glioma cells. However, brain CSC show high CAR expression; hence the infection of CSC occurs also independent from RGD-fiber modification (data not shown). Here we demonstrate efficient viral replication in and cell killing of brain CSC lines by Ad-Delo3-RGD under normoxic and even under hypoxic conditions (Figure 3, 4). OAV like Ad-Delo3-RGD have displayed the potential to efficiently kill not only differentiated cancer cells, but also CSC, including CD44^high^/CD24^low^ cancer breast cells and CD133^high^ glioma CSC [25,26]. However, this is the first report showing that YB-1, which is highly expressed in CSC lines, facilitates adenovirus replication. This is in line with recently published data illustrating that YB-1 is commonly expressed in primary brain CSC and that its expression increased with tumor grade [56].

We next examined the therapeutic anti-tumor efficacy of Ad-Delo3-RGD in an intracranial, orthotopic mouse model using MGMT expressing, TMZ-resistant R28 CSC (Figure 1B). This GBM animal model reflects clinical reality better than using established GBM cell lines. Radiotherapy in combination with the alkylating agent TMZ is currently the standard of care for GBM. GBM expressing MGMT due to an unmethylated MGMT promoter show resistance to treatment with TMZ [57,58]. Furthermore, patients presenting an unmethylated MGMT promoter do not or only marginally benefit from TMZ treatment [59]. Whereas the treatment of R28-GBM bearing mice with TMZ had, as expected no effect, intratumoral injection of Ad-Delo3-RGD significantly prolonged survival of mice. No further increase in survival was observed when Ad-Delo3-RGD injection was combined with TMZ treatment (Figure 6A). This was not unexpected since initial *in vitro* experiments using R28 cells did not indicate any additive or even synergistic inhibition of brain CSC growth when adenoviral infection was combined with TMZ treatment (data not shown). However, the results are in contrast to published data using TMZ resistant melanoma cell of unknown MGMT status [60] or established GBM cell lines [28]. In this context it is mentionable that ionizing radiation (IR) strongly induces YB-1 phosphorylation, enhances repair of DNA double-stranded breaks and affects cell survival [61]. Since current standard of care for patients with GBM includes IR, which is a strong activator of the PI3-K/AKT and MAPK/ERK pathways and promote radio-resistance by activation of the DNA damage response [62,63], it would be interesting or even necessary to include IR in future combinatorial treatment studies.

During treatment of R28-GBM bearing mice, we intratumorally applied 3 x 10^8^ pfu Ad-Delo3-RGD. This is, compared to human, a lower virus load than the well-tolerated dose of the OAV ONYX-015 evaluated for human [64]. Using this dose, microscopic examinations of brain tissues of Ad-Delo3-RGD treated mice showed no signs of inflammation or other related toxicity in adjacent, tumor-surrounding healthy brain, including the subventricular zone, cerebellum and cerebrum, indicating the safety of this YB-1 based virotherapy approach. In addition, our experiments showed, that (i) replication of Ad-Delo3-RGD depends on the presence of YB-1 in cancer stem-like cells, and (ii) Ad-Delo3-RGD only marginally replicates in human immortalized astrocytes (Figure 5). The fact that YB-1 is highly expressed in cancer cells compared to non-neoplastic brain tissue suggests that an YB-1 based virotherapy approach has a high therapeutic index. However, extensive toxicity and biodistribution studies are still necessary to confirm the safety of Ad-Delo3-RGD.

## Conclusion

The results reported here demonstrate that YB-1 is highly expressed in brain cancer stem cell lines and unambiguously, that these cells were efficiently killed by YB-1 dependent OAV *in vitro,* leaving non-neoplastic astrocytes unattached*.* Mean survival of Ad-Delo3-RGD treated R28-bearing mice was significantly longer than that of control mice. To develop new virotherapeutic strategies for GBM, our data are of clinical relevance since it is believed that brain CSC are critical for GBM maintenance and recurrence. In addition, YB-1 expression is linked to multidrug- and radio-resistance and has been repeatedly described to be a predictive biomarker. Even knowing that YB-1 will be upregulated by chemo- as well as radiotherapy, YB-1 analysis of GBM biopsies might improve the therapeutic decision making process in a clinical setting and can help to identify patients who will benefit from novel YB-1 based virotherapy.

## Competing interests

Per S. Holm is CEO and co-founder of XVir Therapeutics GmbH, 80335 Munich, Germany. All other authors declare no conflict of interest.

## Authors’ contributions

PSH, KM and UN conceived and designed the study, performed experiments and analyzed the data. PSH and KM wrote the manuscript. KM, JS, UN, VG, PS performed experiments and analyzed the data. DB, MM and JS provided cell lines and brain tissues. All authors read and approved the final manuscript.
